# Legalization of Cannabis – what’s the impact on mental health?

**DOI:** 10.1192/j.eurpsy.2023.1146

**Published:** 2023-07-19

**Authors:** S. Torres, T. Rocha, J. Leal, J. Moura, A. Lopes

**Affiliations:** 1 Departamento de Psiquiatria e Saúde Mental, Centro Hospitalar Barreiro-Montijo, Lisbon, Portugal

## Abstract

**Introduction:**

With the increasing push to legalize cannabis in Western nations, there is a need to gauge the potential impact of this policy change on vulnerable populations, such as those with mental illness, including schizophrenia, mood and anxiety disorders.

**Objectives:**

Understand the effects of cannabis in people with mental illness and the impact of policies legalizing cannabis in societies.

**Methods:**

Literature review performed on PubMed and Google Scholar databases, using the keywords “cannabis”, “mental health”, “psychiatry”.

**Results:**

Cannabis use is a modifiable risk factor for the development and exacerbation of mental illness. The strongest evidence of risk is for the development of a psychotic disorder, associated with early and consistent use in youth and young adults. Cannabis-related mental health adverse events precipitating Emergency Department (ED) or Emergency Medical Services presentations can include anxiety, suicidal thoughts, psychotic or attenuated psychotic symptoms, and can account for 25–30% of cannabis-related ED visits. Up to 50% of patients with cannabis-related psychotic symptoms presenting to the ED requiring hospitalization will go on to develop schizophrenia. With the legalization of cannabis in various jurisdiction and the subsequent emerging focus of research in this area, our understanding of who (e.g., age groups and risk factors) are presenting with cannabis-related adverse mental health events in an emergency situation is starting to become clearer.

**Conclusions:**

There’s a need to provide a reconciliation of the addiction vulnerability and allostatic hypotheses to explain addiction comorbidity in mentally ill cannabis users, as well as to further aid in developing a rational framework for assessment and treatment of problematic cannabis use in these patients.

**Disclosure of Interest:**

None Declared

